# Supplementary figures and images for: Can the combination of antiplatelet or alteplase thrombolytic therapy with argatroban benefit patients suffering from acute stroke? a systematic review, meta-analysis, and meta-regression
Source: PLoS One. 2024 Feb 27;19(2):e0298226. doi: 10.1371/journal.pone.0298226 (PMC10898750; doi:10.1371/journal.pone.0298226)

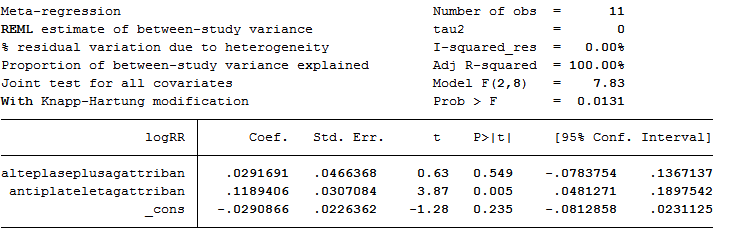

Supplement: S1 Fig — (PNG) [file pone.0298226.s001.png]

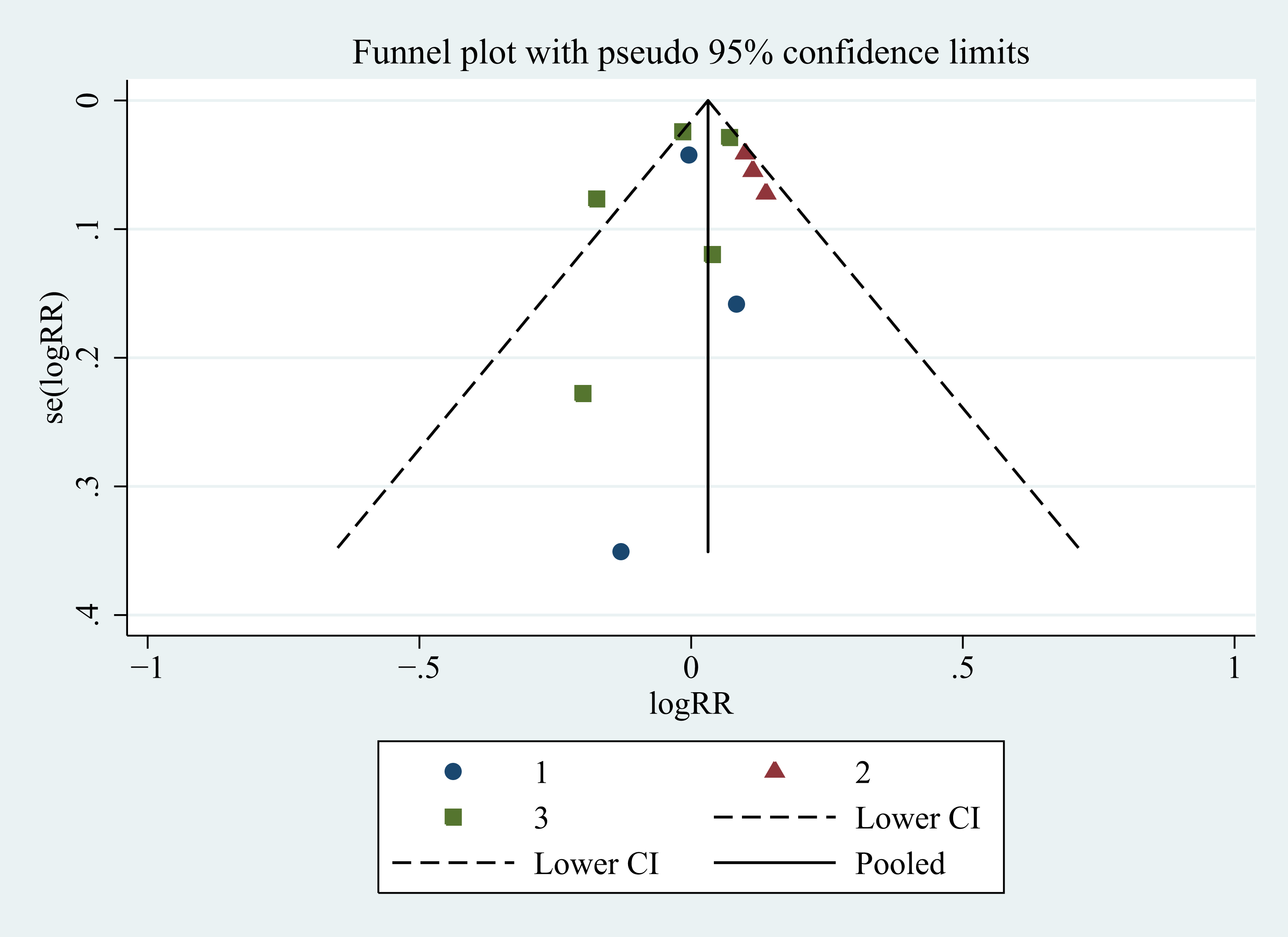

Supplement: S2 Fig — (TIF) [file pone.0298226.s002.tif]

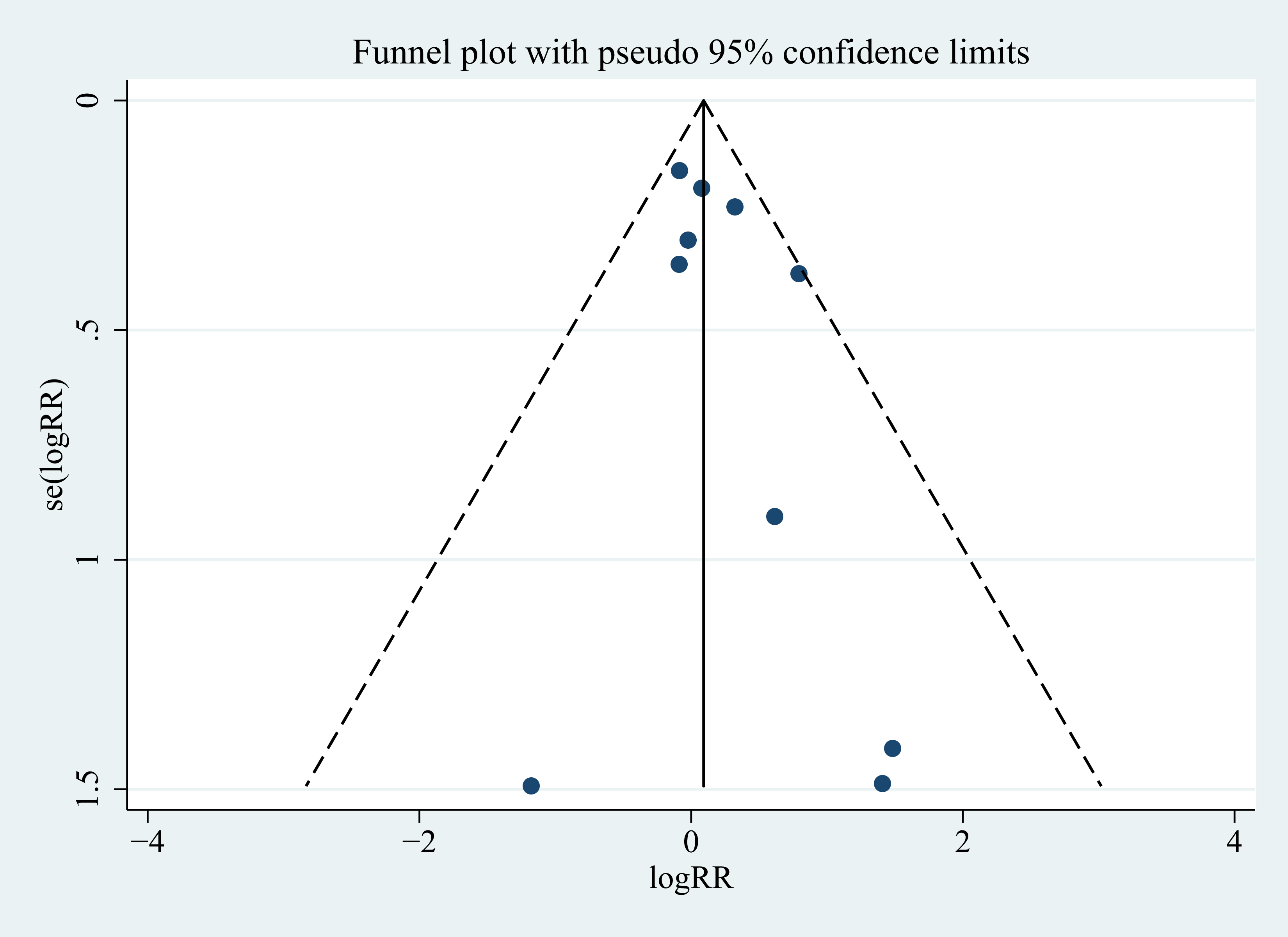

Supplement: S3 Fig — (TIF) [file pone.0298226.s003.tif]
