# Supplementary material for: Can the combination of antiplatelet or alteplase thrombolytic therapy with argatroban benefit patients suffering from acute stroke? a systematic review, meta-analysis, and meta-regression
Source: PLoS One. 2024 Feb 27;19(2):e0298226. doi: 10.1371/journal.pone.0298226 (PMC10898750; doi:10.1371/journal.pone.0298226)
Supplement: S1 Table — (DOC) [file pone.0298226.s004.doc]

**Supplementary Table1 Search engine and query**

| Search engine | Search query | Date of search |
| --- | --- | --- |
| Pubmed (medline) | ("argatroban" [Supplementary Concept]) AND (("Stroke"[Mesh] OR "Brain Ischemia"[Mesh] OR "Cerebral Infarction"[Mesh] OR "Cerebrovascular Disorders"[Mesh])) | 23-April-2 |
| Embase | #1 'argatroban'/exp | 23-April-2 |
|  | #2 'cerebrovascular accident'/exp |  |
|  | #3 'brain ischemia'/exp |  |
|  | #4 'cerebral infarction'/exp OR 'cerebral infarction' OR (cerebral AND ('infarction'/exp OR infarction)) OR 'cerebrovascular disorders':ab,ti |  |
|  | #5 #2 OR #3 OR #4 |  |
|  | #6 #1 AND #5 |  |
| Web of science | TS=(Stroke OR Brain Ischemia OR Cerebral Infarction OR Cerebrovascular Disorders) AND TS=(argatroban) | 23-April-2 |
